# Retrospective Analysis of the Impact of Cetylpyridinium Chloride on Oral Healthcare in Patients Wearing Removable Orthodontic Appliances

**DOI:** 10.3290/j.ohpd.c_1801

**Published:** 2025-01-08

**Authors:** Zhuqing Yu, Xiaoteng Shen

**Affiliations:** a Zhuqing Yu Dentist, Department of Stomatology, Hangzhou Fuyang Hospital of Traditional Chinese Medicine, Hangzhou, Zhejiang, China. Designed the study, performed the research, analysed the data, contributed new methods, wrote the paper, approved final version of manuscript.; b Xiaoteng Shen Dentist, Physical Examination Center, Hangzhou Fuyang Hospital of Traditional Chinese Medicine, Hangzhou, Zhejiang, China. Designed the study, performed the research, analysed the data, approved final version of manuscript. Both authors contributed equally to this work and should be considered equal first authors.

**Keywords:** cetylpyridinium chloride, inflammatory cytokines, oral healthcare, removable orthodontic appliance

## Abstract

**Purpose:**

To examine the alterations in oral healthcare indicators subsequent to the administration of cetylpyridinium chloride.

**Materials and Methods:**

In this retrospective study, clinical data of 58 patients who received orthodontic treatment using removable appliances at our medical facility were collected. Patients were divided into two groups based on whether they used cetylpyridinium chloride during orthodontic treatment: the combined group (n = 31, received 0.1% cetylpyridinium chloride gargle in addition to periodontal cleaning during the use of orthodontic appliances, with gargling applied three times daily for at least 1 min after meals) and the cleaning group (n = 27, received only periodontal cleaning). Data on oral healthcare were collected and analysed at 1, 3, and 6 months into the treatment regimen. The indices evaluated were gingival index (GI), sulcus bleeding index (SBI), probing depth (PD), and plaque index (PLI).

**Results:**

Subsequent evaluations revealed that, at 3 and 6 months post-intervention, patients in the intervention group exhibited statistically lower scores in GI, SBI, and PLI when compared to the control group. Similarly, the PD measurements showed more statistically significant reductions at each follow-up interval — 1, 3, and 6 months — in the intervention group. IL-10 levels were notably higher in the intervention group at 6 months.

**Conclusion:**

Integrating cetylpyridinium chloride into the oral healthcare regimen for patients using removable orthodontic appliances has been shown to statistically significantly improve oral health, enhance periodontal functions, and reduce inflammatory responses in the gingival sulcus.

Orthodontic methods are mainly employed in clinical settings for the correction of malocclusions and are favoured by orthodontists for their straightforward applicability and pronounced efficacy.^16^ Orthodontic braces, fundamental instruments in these interventions, are designed to amend malformations in the frontal bone, misaligned teeth, and supporting periodontal structures through the application of forces or the functional dynamics of masticatory and perioral muscles.^20^ This aids in the normal development and maturation of the dentition and jaws.

However, with the widespread adoption of orthodontic appliances, their disadvantages have become increasingly apparent. Notably, the attachment of these devices often detrimentally impacts the health of periodontal tissues. Some studies have even highlighted that orthodontic appliances may inflict minor damage to these tissues.^17^ Issues such as plaque accumulation during orthodontic treatment can lead to frequent instances of gingival redness, hyperplasia, bleeding upon probing, and in severe cases, the loss of attachment and alveolar bone resorption.^3,13^ Consequently, how to enhance the oral healthcare of patients undergoing orthodontic treatment and maintain optimal oral hygiene have become important challenges.

Cetylpyridinium chloride, a surfactant known for its ability to decrease surface tension, thereby inhibiting and exterminating bacteria, has shown promise in addressing these issues. In-vitro studies have validated its efficacy in eradicating various oral pathogens.^11^ Moreover, the use of cetylpyridinium chloride as a gargle has been found to prevent plaque formation, maintain oral cleanliness, and eliminate oral odours.^5^ This retrospective analysis aimed to explore how the integration of a cetylpyridinium chloride gargle regimen in patients with removable orthodontic appliances can enhance oral healthcare and reduce the levels of inflammatory markers in the gingival crevicular fluid.

## MATERIALS AND METHODS

### Study Design and Patients

This study was conducted under the approval of the Hangzhou Fuyang Hospital of Traditional Chinese Medicine’s ethics committee, employing a retrospective study design. Utilising the hospital’s electronic medical records system, patients who underwent orthodontic treatment with removable orthodontic appliances, including clear aligners, between January 2020 and October 2023 were identified and screened based on specific inclusion and exclusion criteria, initially including 98 cases.

Inclusion criteria: (1) Patients undergoing orthodontic treatment with a removable orthodontic appliance. (2) No apparent dental caries, or carious lesions that had been adequately restored. (3) No concurrent periodontal diseases such as periodontal trauma or atrophy. (4) No missing teeth, except for the third molars. (5) No systemic diseases. (6) Comprehensive baseline data, including gender, age, and oral healthcare indicators (gingival index [GI], sulcus bleeding index [SBI], probing depth [PD], plaque index [PLI]) were recorded before and during orthodontic treatment at intervals of 1 month, 3 months, and 6 months. Levels of inflammatory cytokines interleukin-6 (IL-6) and interleukin-10 (IL-10) in the gingival crevicular fluid were measured before the commencement and at 6 months into the orthodontic treatment, and a thorough assessment of clinical outcomes was conducted post-orthodontic treatment.

Exclusion Criteria: (1) Any antibiotic or hormonal treatment within the 3 months preceding the study inclusion. (2) Systemic periodontal treatment received within the 3 months prior to the intervention. (3) Presence of extensive poor restorations within the oral cavity. (4) Women who were pregnant or lactating during the study period.

Following the predefined criteria, 40 of the 98 patients were excluded, and 58 patients were retrospectively selected as the study subjects and subsequently allocated into two groups according to different treatment methods. The cleaning group (n =27) received periodontal maintenance involving ultrasonic scaler cleaning at 1, 3, and 6 months of orthodontic treatment, in addition to regular toothbrushing. The combined group (n = 31) underwent periodontal cleaning supplemented with a cetylpyridinium chloride gargle, which was used 3x daily for at least 1 min after meals (0.1% cetylpyridinium chloride gargle; Nanjing Hencer Pharmaceutical) in conjunction with their periodontal maintenance throughout the duration of wearing their orthodontic appliances. The demographic breakdown in the combined group included 20 females and 11 males, with an average age of 22.97 ± 6.05 years. The cleaning group comprised 19 females and 8 males, with an average age of 23.19 ± 4.89 years. Statistical analysis revealed no statistically significant differences in the general clinical data between the two groups (p>0.05), confirming that the groups were comparable and well-matched for the purposes of this study.

### Data Collection

Based on the following formula for sample size calculation, and in conjunction with data from the literature, the efficacy rate of the combined intervention group was anticipated to be 90%, while that of oral cleaning was expected to be 80%. With an efficacy threshold of Δ = 0, α = 0.025, β = 0.10, and a 5% dropout rate taken into account, the sample size (N) for each group was calculated to be 25 cases:


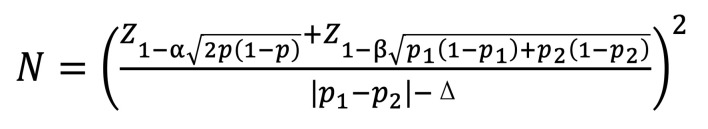


Utilising the hospital’s electronic medical records system, data were systematically collected on the following parameters: baseline clinical data (gender, age), oral healthcare indicators (gingival index [GI index], sulcus bleeding index [SBI index], probing depth [PD], plaque index [PLI index]) both prior to and during orthodontic treatment (at 1 month, 3 months, and 6 months). Additionally, levels of the inflammatory cytokines interleukin-6 (IL-6) and interleukin-10 (IL-10) were measured in the gingival crevicular fluid before the commencement of orthodontic treatment and at 6 months post-treatment. Clinical outcomes following orthodontic treatment were also evaluated. One data collector had received coding-related training (including how to review variables and use data extraction forms), and one member of the research team had been assigned to provide continuous supervision of the data collector to ensure that the data extraction process was accurate, consistent, and objective.

The GI index was assessed using the criteria proposed by Löe and Silness,^14^ which categorises gingival health into four grades: grade 0 indicates healthy gingiva; grade 1, mildly inflamed gingiva; grade 2, moderately inflamed gingiva; and grade 3, severely inflamed gingiva. The SBI index^25^ classifies gingival health into six grades ranging from 0 (perfectly healthy) to 5 (significant gingival swelling and colour change). Probing depth (PD)^15^ measures the distance from the gingival margin to the base of the periodontal or gingival pocket. The PLI index, according to Silness and Löe,^22^ differentiates four grades of plaque accumulation: grade 0 indicates no plaque at the gingival margin; grade 1, a thin film of plaque at the gingival margin; grade 2, a moderate amount of plaque at the gingival margin or adjacent surfaces; and grade 3, a large accumulation of soft tartar in the gingival sulcus or at the gingival margin.

Additionally, measurements of IL-6 and IL-10 levels were conducted through enzyme-linked immunosorbent assays (ELISA) using kits from Thermo Fisher Scientific (Waltham, MA, USA). The collection of gingival crevicular fluid was performed using the intragroove filter-paper strip method. Prior to periodontal cleaning, specialised Whatman Grade 3 filter-paper strips (Shanghai Bestest Biological Technology) were gently inserted into the buccal mesial or distal periodontal pocket or gingival sulcus of the patient until slight resistance was felt. After 30 s of collection, the strips were removed and sealed in sterile membranes, placed into EP tubes, and stored at -80°C until use. When needed for testing, the filter paper strips were thawed at 4°C for 1 h and then subjected to ultracentrifugation at 10,000 rpm for 10 min. The supernatant was collected and analysed using ELISA.7

Clinical outcomes were evaluated six months post-treatment. The effectiveness of orthodontic treatment was categorised into three levels: markedly effective (where patients’ teeth were well-aligned and functional), effective (where there was statistically significant improvement in periodontal function, though some tooth deformities in patients had not been adequately improved), and ineffective (where the treatment failed to meet the above criteria). The cumulative count of successful outcomes included both markedly effective and effective cases.

### Quality Control

To ensure the accuracy of data collection and entry, a two-person team was responsible for screening patients, gathering data, and entering it into the system. The individuals assigned to data entry were members of the medical and nursing staff from the dental department, who possess specialised knowledge and expertise in dentistry.

### Outcome Measures and Statistical Analysis

The anticipated outcome of this study is that the incorporation of cetylpyridinium chloride gargle in the treatment regimen for patients using removable orthodontic appliances will enhance oral healthcare and reduce the levels of inflammatory markers in the gingival crevicular fluid.

Data analysis was conducted using SPSS version 21.0 (IBM; Armonk, NY, USA). The collected were normally distributed and presented as mean ± standard deviation. Group comparisons were made using the t-test for continuous variables. Categorical data were expressed in percentages and analysed using the chi-squared test. Statistical significance was established at a p-value < 0.05.

## RESULTS

### Comparison of Oral Healthcare Indicators

**Fig 1:**
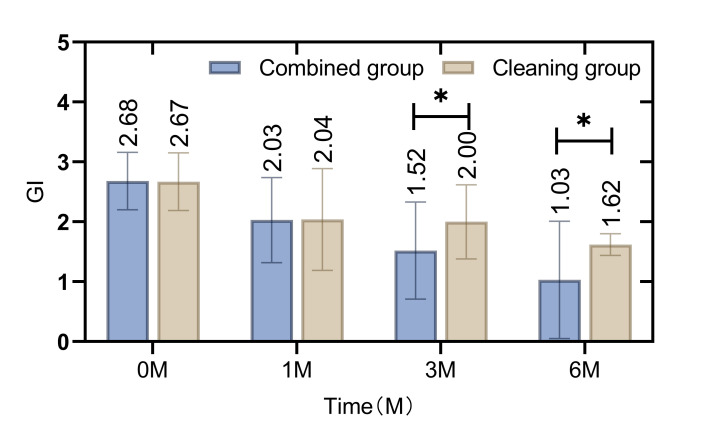
Difference in GI index between patients in the combined group and the cleaning group before and after the intervention. The GI index was lower in the combined group than in the cleaning group at 3 and 6 months after the intervention (p < 0.05).

**Fig 2:**
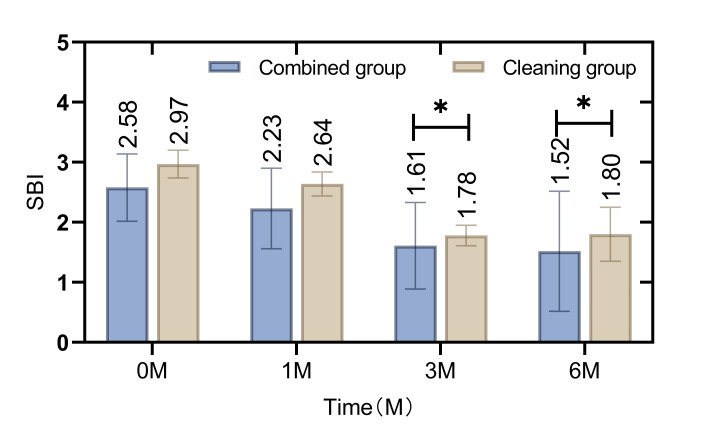
Difference in SBI index before and after intervention between patients in the combined group and the cleaning group. SBI index was lower in the combined group than in the cleaning group at 3 and 6 months after intervention (p < 0.05).

**Fig 3:**
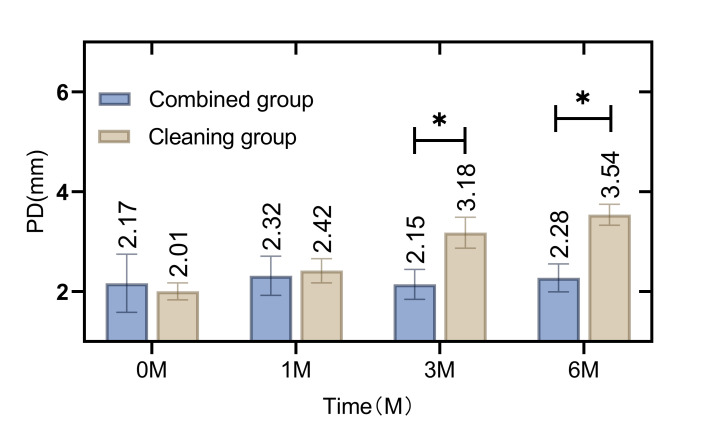
Difference in PD between patients in the combined group and the cleaning group before and after intervention. At 1 month, 3 months and 6 months after intervention, the PD of the combined group was statistically significantly lower than that of the cleaning group (p < 0.05).

**Fig 4:**
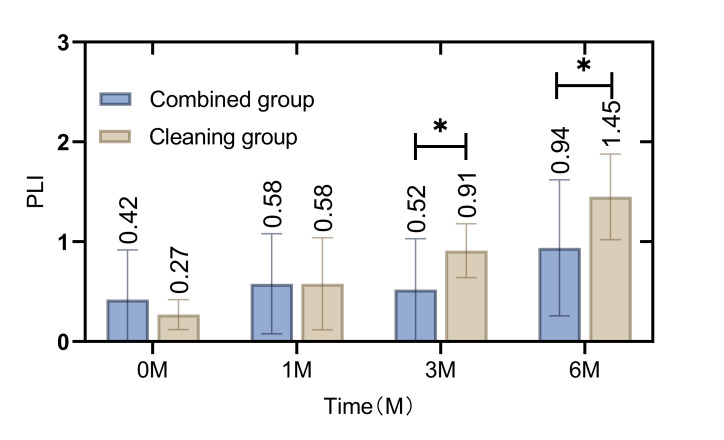
Difference in PLI scores between patients in the combined group and the cleaning group before and after intervention. At 3 months and 6 months after intervention, PLI scores in the combined group were statistically significantly lower than those in the cleaning group (p < 0.05).

In the initial assessment, there was no statistically significant difference in oral healthcare indicators such as the GI (Fig 1), SBI (Fig 2), PD (Fig 3), and PLI (Fig 4) between the patients in the combined group and those in the cleaning group (p = 0.865, 0.162, 0.269, 0.338). However, following the intervention, notable improvements were observed in the combined group. Specifically, the GI, SBI, and PLI scores were statistically significantly lower in the combined group than in the cleaning group at 3 and 6 months post-intervention (p = 0.009, 0.000, p = 0.000, 0.000, p = 0.000, 0.000 ). Similarly, the depth of PD was consistently lower in the combined group than in the cleaning group at 1 month, 3 months, and 6 months after the intervention (p = 0.008, 0.026, 0.018).

### Comparison of Inflammatory Factor Levels in Gingival Crevicular Fluid

**Fig 5:**
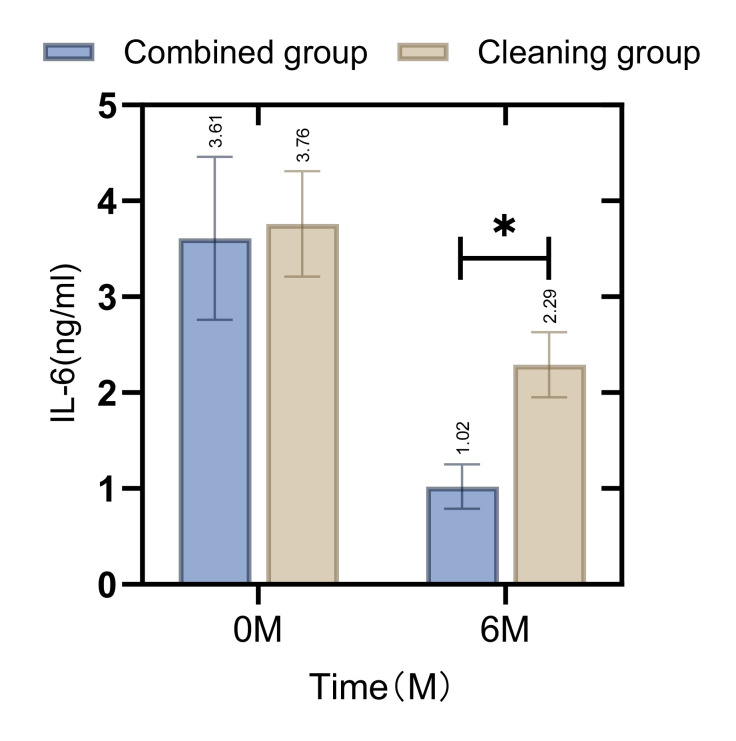
Comparison of L-6 levels in gingival crevicular fluid between patients in the combined group and the cleaning group before and after the intervention. The difference in the gingival crevicular fluid IL-6 levels between the groups before the intervention was not statistically significant (p>0.05), and the gingival crevicular fluid IL-6 levels in the combined group were lower than those of the cleaning group after 6 months (p < 0.05). *Indicates that the differences between groups for the same indicator are statistically significant.

**Fig 6:**
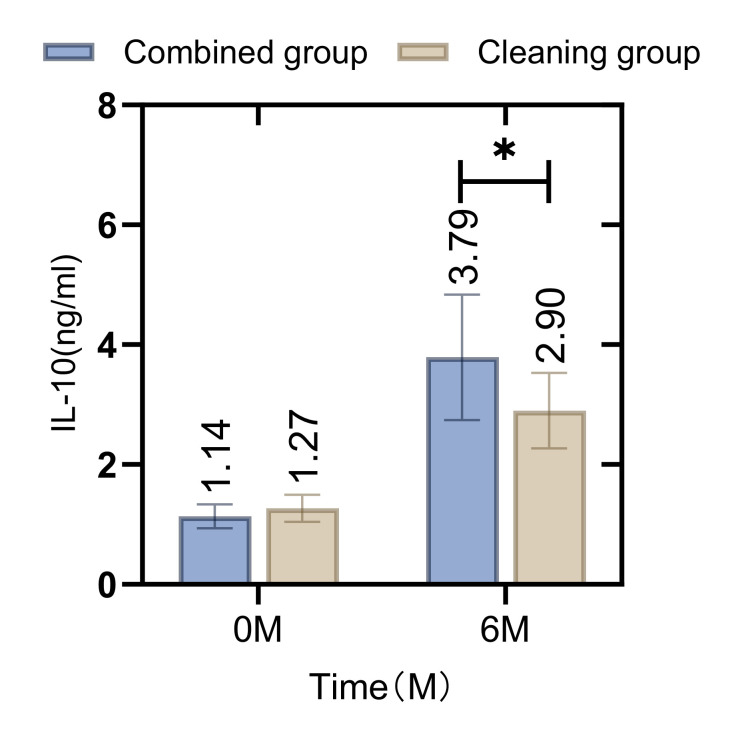
Comparison of IL-10 levels in gingival crevicular fluid between patients in the combined group and the cleaning group before and after intervention. The difference in IL-10 levels in gingival crevicular fluid between the groups before intervention was not statistically significant (p < 0.05), and the IL-10 level in the combined group was higher than that of the cleaning group after 6 months (p < 0.05). *Indicates that the differences between groups for the same indicator are statistically significant.

Before the intervention, there was no statistically significant difference in the levels of IL-6 and IL-10 in the gingival crevicular fluid between the combined and cleaning groups (p = 0.114, 0.683). However, at the 6-month follow-up, the levels of IL-6 in the combined group were found to be lower and the levels of IL-10 were higher compared to those in the cleaning group. These differences were statistically significant (p = 0.021, 0.012), as illustrated in Figs 5 and 6.

### Comparison of Clinical Effects Between Patients in the Combined Group vs the Cleaning Group

The combined group showed varied levels of treatment effectiveness. Specifically, there were 18 cases classified as markedly effective, 11 as effective, and 2 as ineffective. This resulted in a total effective rate of 93.55% for the combined group. In comparison, the cleaning group achieved an overall effectiveness rate of 88.89%. The statistical analysis indicated that the difference in effectiveness between the two groups was not statistically significant (p = 0.528).

## DISCUSSION

In 1976, Cohen et al^6^ introduced a patient-centered standard for oral health evaluation known as the oral health-related quality of life (OHRQoL). This standard posits that oral health is intricately linked to various facets of an individual’s life, including socio-emotional health, self-image, well-being, expectations, and satisfaction.^6^ The World Health Organization has recognised the adoption of this standard as a pivotal component of the Global Oral Health Program, affirming its significance in contemporary dental research.

In recent years, as the overall quality of life has improved, individuals have increasingly acknowledged the impact of oral diseases on their lives, particularly in terms of social identity and personal well-being. Facial defects and functional impairments, influenced by social activities and work interactions, can significantly impact the health and psychological well-being of adults.^12^ Research has shown that patients with malocclusion, such as those with missing teeth, are less confident than their peers and have diminished physical function and life abilities.^24^ Additionally, studies indicate that the higher an individual’s confidence in their facial appearance, the more likely they are to exhibit extroverted and emotionally stable personality traits; conversely, lower confidence correlates with introversion and emotional instability, underscoring the importance of proactive early orthodontic treatment.^4^

Although removable orthodontic appliances offer greater flexibility compared to their fixed counterparts, they too are susceptible to promoting adverse oral health effects. Such negative outcomes are often attributed to the propensity of these appliances to accumulate food particles and plaque, which can subsequently compromise the efficacy of orthodontic treatment. At the same time, personal oral hygiene can also greatly affect the use of orthodontic appliances. Previous studies have indicated that those who wear orthodontic appliances but neglect oral hygiene face a statistically significantly higher risk of swelling, gingivitis, and even carious lesions.^8^ This underscores the critical importance of enhancing oral hygiene interventions for patients using orthodontic appliances.

The current study employed a retrospective analysis to evaluate the clinical efficacy of integrating cetylpyridinium chloride as an adjunctive treatment in patients wearing removable orthodontic appliances. The findings indicate that the combined group, which augmented their regimen with daily cetylpyridinium chloride gargle, exhibited statistically significant improvements in oral healthcare at 3 and 6 months into the orthodontic treatment. Key indicators such as the GI, SBI, PD, and PLI were notably enhanced. Of particular interest was the reduction in the depth of PD, which was statistically significantly lower in the combined group than in the cleaning group from the first month of treatment onward. This suggests that the intervention with cetylpyridinium chloride gargle improves oral health in patients using non-fixed orthodontic appliances.^19^

The authors of this study posit that orthodontic treatment is not only pivotal for correcting dental irregularities but also represents an efficacious approach to managing such conditions. However, the extended presence of orthodontic appliances in the oral cavity can facilitate the accumulation of food residues and plaque, consequently altering the oral environment and potentially precipitating conditions such as gingivitis and periodontitis.^21^ Traditional toothbrushing predominantly targets plaque and food debris on the tooth surfaces but often fails to eliminate bacteria and food remnants lodged in the crevices and concealed areas of the teeth. While ultrasonic periodontal cleaning effectively removes plaque and soft tartar from between the teeth, in the grooves and along the gingival sulcus, its efficacy heavily relies on instrumentation and patient compliance, which can sometimes be suboptimal.^10^

The principal active component in cetylpyridinium chloride gargle, cetylpyridinium chloride, adheres to the surface of bacterial cell membranes and interacts with the anions in the cell wall. This interaction increases the permeability of the cell wall and induces the denaturation of bacterial proteins, thereby exerting a bactericidal effect.^23^ Current studies^2^ have demonstrated that using a 0.1% cetylpyridinium chloride gargle can effectively inhibit plaque formation and reduce the clinical symptoms associated with gingivitis. In this study, no statistically significant differences in oral health indicators were observed between the combined group and the cleaning group at one month of orthodontic treatment. This finding could be attributed to the fact that periodontal cleaning initially removes most of the plaque, calculus, and soft tartar, and the formation of new plaque takes time. Consequently, the effects of the cetylpyridinium chloride gargle may not be immediately apparent. Improvements in oral healthcare indicators were noted at 3 and 6 months in the combined group, likely due to the ongoing use of the cetylpyridinium chloride gargle. However, we believe that long-term use of cetylpyridinium chloride gargle may pose certain risks, such as disrupting the balance of oral microbiota, triggering oral ulcers, staining the teeth and mucous membranes, and diminishing taste sensitivity. Therefore, it is recommended that patients adhere strictly to the prescribed dosage and usage guidelines to prevent the emergence of new oral healthcare issues.^3^

Further analysis revealed differences in the levels of inflammatory factors in the gingival crevicular fluid between the two groups post-treatment. Notably, IL-6 levels were lower and IL-10 levels were higher in the combined group compared to the cleaning group. IL-6 is known to promote the expression and transcription of various inflammatory factors, exacerbating local inflammation, whereas IL-10 has anti-inflammatory properties that can ameliorate the clinical symptoms of periodontitis.^9^ Research on the use of cetylpyridinium chloride in patients with periodontitis^1^ has indicated that this intervention can reduce levels of inflammatory factors such as TNF-α and MMP-9 in the gingival sulcus, corroborating the findings of this study. These reductions are indicative of improved oral healthcare.^18^ However, the differences in clinical outcomes between the two groups were not statistically significant. This may be attributed to the short follow-up period and the fact that while the cetylpyridinium chloride gargle can enhance oral healthcare, the clinical effectiveness of orthodontic treatment largely depends on the proper use and standardisation of the orthodontic appliances.

## CONCLUSION

Integrating cetylpyridinium chloride into the treatment regimen for patients wearing removable orthodontic appliances has demonstrated efficacy in enhancing oral healthcare. This intervention statistically significantly improved oral health and periodontal function, while also reducing the levels of inflammatory markers in the gingival crevicular fluid. A key innovation of this study lies in the validation of cetylpyridinium chloride gargle’s effectiveness in improving the oral health of patients undergoing treatment with removable orthodontic appliances. This was accomplished through meticulous follow-up and by comparing the changes in inflammatory factor levels within the gingival sulcus.

However, the study is not without limitations. Notably, it features a relatively small sample size and a brief follow-up period, which may limit the generalisability of the findings. Additionally, the absence of a multicenter approach restricts the broader applicability of the results. These limitations are recognised as critical areas for improvement and will be addressed in future research to strengthen the findings and expand their relevance.
